# The putative role of the epipeptide EpeX in *Bacillus subtilis* intra-species competition

**DOI:** 10.1099/mic.0.001344

**Published:** 2023-06-08

**Authors:** Margarita Kalamara, James Abbott, Tetyana Sukhodub, Cait MacPhee, Nicola R. Stanley-Wall

**Affiliations:** ^1^​ Division of Molecular Microbiology, School of Life Sciences, University of Dundee, Dundee, DD5 4EH, UK; ^2^​ Data Analysis Group, Division of Computational Biology, School of Life Sciences, University of Dundee, Dundee, DD5 4EH, UK; ^3^​ National Biofilms Innovation Centre, School of Physics & Astronomy, University of Edinburgh, EH9 3FD Edinburgh, UK

**Keywords:** *Bacillus subtilis*, biofilm, EpeX, epipeptide, kin discrimination

## Abstract

Bacteria engage in competitive interactions with neighbours that can either be of the same or different species. Multiple mechanisms are deployed to ensure the desired outcome and one tactic commonly implemented is the production of specialised metabolites. The Gram-positive bacterium *Bacillus subtilis* uses specialized metabolites as part of its intra-species competition determinants to differentiate between kin and non-kin isolates. It is, however, unknown if the collection of specialized metabolites defines competitive fitness when the two isolates start as a close, interwoven community that grows into a densely packed colony biofilm. Moreover, the identity of specialized metabolites that have an active role in defining the outcome of an intra-species interaction has not been revealed. Here, we determine the competition outcomes that manifest when 21 environmental isolates of *B. subtilis* are individually co-incubated with the model isolate NCIB 3610 in a colony biofilm. We correlated these data with the suite of specialized metabolite biosynthesis clusters encoded by each isolate. We found that the *epeXEPAB* gene cluster was primarily present in isolates with a strong competitive phenotype. This cluster is responsible for producing the epipeptide EpeX. We demonstrated that EpeX is a competition determinant of *B. subtilis* in an otherwise isogenic context for NCBI 3610. However, when we competed the NCIB 3610 EpeX-deficient strain against our suite of environmental isolates we found that the impact of EpeX in competition is isolate-specific, as only one of the 21 isolates showed increased survival when EpeX was lacking. Taken together, we have shown that EpeX is a competition determinant used by *B. subtilis* that impacts intra-species interactions but only in an isolate-specific manner.

## Introduction

Specialized metabolites (also known as secondary metabolites) are a diverse class of bioactive molecules. These molecules, while classed as ‘secondary’, have a wide range of crucial roles for bacterial life, ranging from nutrient acquisition (e.g. siderophores [1]) to regulating social behaviours [2] and impacting competition with microbes in the surrounding environment [3]. Specialized metabolites shape the social interactions of single isolates, intra-species, and inter-species communities.

*Bacillus subtilis* is a Gram-positive soil-dwelling bacterium known for its ability to produce a wide range of specialized metabolites, with approximately 4 % of its genome encoding the biosynthetic machinery needed for their production [4]. The wide-ranging diversity of molecules produced is, in part, the reason behind the wide use of *B. subtilis* and closely related species as biocontrol agents, as various specialized metabolites have antimicrobial properties [5]. In addition to facilitating inter-species competition, many of the specialized metabolites produced by *B. subtilis* play crucial roles in regulating the development of biofilms and spores in single-isolate *B. subtilis* communities [2, 6–8].

Interactions between different isolates of *B. subtilis* are an additional aspect of social interactions that are impacted by specialized metabolites. Some specialized metabolites, such as sporulation killing factor, subtilosin A, bacillaene, and sublancin 168 are important kin discrimination determinants for *B. subtilis *, as defined using swarm meeting assays, where swarms of different isolates are moving towards each other on semi-solid media plates [9]. This role of specialized metabolites in intra-species interactions has been further supported by looking at the inhibitory properties of different isolates in so-called ‘halo formation’ assays, where a focal strain is grown in a colony on top of a lawn of the target strain on LB plates. While no molecular work was done, a correlation between isolates encoding different biosynthetic gene clusters and competition was drawn. This correlation was however not perfect, such that in some cases isolates encoding the same suite of biosynthetic gene clusters would still inhibit each other [10]. These analyses highlight the complexities in elucidating the mechanisms driving competition outcomes and the complexities in defining the outcome of intra-species interactions.

In this work, we were interested in understanding the molecules that govern the competitive dynamics of isolates growing within the same niche, a mixed isolate colony biofilm. In *B. subtilis *, competitive fitness in a spatially constrained mixed community is known to be impacted by the spatial arrangement of the founding cells [11, 12] and by the presence of polymorphic toxins [13]. However, knowledge surrounding the role that specialized metabolites play in shaping these interactions in mixed communities is lacking. Here, to address this knowledge gap, we set out to explore the relationship between the suite of specialized metabolite biosynthesis clusters (SMBCs) encoded by 21 soil isolates of *B. subtilis* and the model isolate NCIB 3610 and their pairwise competitive fitness within colony biofilms. The 21 soil isolates were isolated from soil and compost samples that were provided by citizens living in the area surrounding Dundee, UK [14]. We obtained complete whole-genome sequence data and detected the SMBC within all 22 genomes. We next correlated the presence of the accessory SMBCs with the competitive fitness of the isolates relative to the model isolate NCIB 3610. We identified that the SMBC whose presence most closely correlated with a strong competitive phenotype was the *epeXEPAB* cluster, which is responsible for the production of the epipeptide EpeX. EpeX is an antimicrobial that is known to induce cell-envelope stress response and can kill *B. subtilis* when applied extrinsically via membrane perturbations [15–17]. The locus responsible for the production of this antimicrobial peptide is widely distributed among firmicutes and expression of the operon is under the control of the regulators AbrB and Spo0A [18]. We explored the role of EpeX in competitive fitness by constructing a deletion mutant of the biosynthetic cluster in the model isolate NCIB 3610. We found that, in an otherwise isogenic context, EpeX is an important determinant of competitive fitness, with the strain encoding the cluster occupying a higher proportion of the mixed community when compared with the NICB 3610 EpeX-deficient mutant. However, when testing the generality of EpeX as an intra-species competition determinant, we only identified one isolate within our suite of 21 isolates that exhibited increased survival when competed with the EpeX-deficient strain of NCIB 3610 rather than the NCIB 3610 parental strain. Additionally, when exploring the role that EpeX has as a competition determinant in other isolates, we found that the absence of the *epeXEPAB* cluster does not impact competitive fitness in two other soil isolates. In combination, our results reveal that EpeX affects intra-species competition outcomes in a highly isolate-specific manner.

## Methods

### Growth conditions and strains used

All strains used in this study are listed in Table 1. For routine growth of *Bacillus subtilis* and *Escherichia coli* strains, lysogeny broth liquid media was made using the following recipe: 1 % (w/v) Bacto-peptone, 1 % (w/v) NaCl, 0.5 % (w/v) yeast extract. For solid plates, LB broth was supplemented with 1.5 % (w/v) agar. LB media was sterilized by autoclaving. When necessary, LB media cultures and plates were supplemented with antibiotics at the following concentrations for *B. subtilis *: 10 µg ml^−1^ kanamycin, 100 µg ml^−1^ spectinomycin and 5 µg ml^−1^ chloramphenicol. For growth of *E. coli* carrying plasmids of interest, the LB plates and liquid media were supplemented with 100 µg ml^−1^ of ampicillin, or 25 µg ml^−1^ chloramphenicol as required. Single isolate biofilm and mixed isolate biofilm competition assays were conducted using MSgg (Minimal Salts glycerol glutamate) media. MSgg was made by first making a base medium, consisting of 5 mM potassium phosphate, 100 mM MOPS at pH 7.0, supplemented with 1.5 % (w/v) agar. The media base was autoclaved and cooled to 55 °C. The base medium was supplemented with 2 mM MgCl_2_, 700 µM CaCl_2_, 50 µM FeCl_3_, 50 µM MnCl_2_, 1 µM ZnCl_2_, 2 µM thiamine, 0.5 % (v/v) glycerol and 0.5 % (w/v) glutamic acid. A volume of 23 ml of MSgg melted media was added to each 9 cm diameter petri dish and the plates were solidified at room temperature. The surface of the solid plates was dried for 1 h under a laminar flow cabinet prior to use in experiments.

**Table 1.** Strains used in this study

Strain	Code name*	Genotype†	Source‡
NCIB 3610		Wild-type	B.G.S.C.
168		*trpC2*	B.G.S.C.
NRS6220	NRS6103g	NRS6103 *amyE::Phy-spank-gfp mut2 (cml*)	pBL165 into NRS6103
NRS6221	NRS6105g	NRS6105 *amyE::Phy-spank-gfp mut2 (cml*)	pBL165 into NRS6105
NRS6222	NRS6153g	NRS6153 *amyE::Phy-spank-gfp mut2 (cml*)	pBL165 into NRS6153
NRS6223	NRS6096g	NRS6096 *amyE::Phy-spank-gfp mut2 (cml*)	pBL165 into NRS6096
NRS6881	NRS6085g	NRS6085 *amyE::Phy-spank-gfp mut2 (cml*)	pBL165 into NRS6085
NRS6882	NRS6099g	NRS6099 *amyE::Phy-spank-gfp mut2 (cml*)	pBL165 into NRS6099
NRS6883	NRS6107g	NRS6107 *amyE::Phy-spank-gfp mut2 (cml*)	pBL165 into NRS6107
NRS6884	NRS6116g	NRS6116 amyE::Phy-spank-gfp mut2 (cml)	pBL165 into NRS6116
NRS6885	NRS6118g	NRS6118 *amyE::Phy-spank-gfp mut2 (cml*)	pBL165 into NRS6118
NRS6886	NRS6121g	NRS6121 *amyE::Phy-spank-gfp mut2 (cml*)	pBL165 into NRS6121
NRS6887	NRS6127g	NRS6127 *amyE::Phy-spank-gfp mut2 (cml*)	pBL165 into NRS6127
NRS6888	NRS6128g	NRS6128 *amyE::Phy-spank-gfp mut2 (cml*)	pBL165 into NRS6128
NRS6889	NRS6132g	NRS6132 *amyE::Phy-spank-gfp mut2 (cml*)	pBL165 into NRS6132
NRS6890	NRS6145g	NRS6145 *amyE::Phy-spank-gfp mut2 (cml*)	pBL165 into NRS6145
NRS6891	NRS6160g	NRS6160 *amyE::Phy-spank-gfp mut2 (cml*)	pBL165 into NRS6160
NRS6892	NRS6181g	NRS6181 *amyE::Phy-spank-gfp mut2 (cml*)	pBL165 into NRS6181
NRS6893	NRS6183g	NRS6183 *amyE::Phy-spank-gfp mut2 (cml*)	pBL165 into NRS6183
NRS6894	NRS6186g	NRS6186 *amyE::Phy-spank-gfp mut2 (cml*)	pBL165 into NRS6186
NRS6895	NRS6187g	NRS6187 *amyE::Phy-spank-gfp mut2 (cml*)	pBL165 into NRS6187
NRS6896	NRS6190	NRS6190 *amyE::Phy-spank-gfp mut2 (cml*)	pBL165 into NRS6190
NRS6897	NRS6202g	NRS6202 *amyE::Phy-spank-gfp mut2 (cml*)	pBL165 into NRS6202
NRS6931		168 *amyE::Phy-spank-mTagBFP* [19]	pNW2304 into 168
NRS6932	NCIB 3610b	NCIB 3610 *amyE::Phy-spank-mTagBFP* [19]	NRS6931 SPP1 into NCIB 3610
NRS6900		168 *amyE::Phy-spank-gfp mut2 (cml*)	pBL165 into 168
NRS6942	NCIB 3610 g	NCIB 3610 *amyE::Phy-spank-gfp mut2 (cml*)	NRS6900 SPP1 into NCIB 3610
NRS6934	NRS6096b	NRS6096 *amyE::Phy-spank-mTagBFP* [19]	pNW2304 into NRS6096
NRS6935	NRS6103b	NRS6103 *amyE::Phy-spank-mTagBFP* [19]	pNW2304 into NRS6103
NRS6936	NRS6105b	NRS6105 *amyE::Phy-spank-mTagBFP* [19]	pNW2304 into NRS6105
NRS6937	NRS6118b	NRS6118 *amyE::Phy-spank-mTagBFP* [19]	pNW2304 into NRS6118
NRS6938	NRS6153b	NRS6153 *amyE::Phy-spank-mTagBFP* [19]	pNW2304 into NRS6153
NRS6943	NRS6085b	NRS6085 *amyE::Phy-spank-mTagBFP* [19]	pNW2304 into NRS6085
NRS6944	NRS6099b	NRS6099 *amyE::Phy-spank-mTagBFP* [19]	pNW2304 into NRS6099
NRS6945	NRS6107b	NRS6107 *amyE::Phy-spank-mTagBFP* [19]	pNW2304 into NRS6107
NRS6946	NRS6116b	NRS6116 *amyE::Phy-spank-mTagBFP* [19]	pNW2304 into NRS6116
NRS6947	NRS6121b	NRS6121 *amyE::Phy-spank-mTagBFP* [19]	pNW2304 into NRS6121
NRS6948	NRS6127b	NRS6127 *amyE::Phy-spank-mTagBFP* [19]	pNW2304 into NRS6127
NRS6949	NRS6128b	NRS6128 *amyE::Phy-spank-mTagBFP* [19]	pNW2304 into NRS6128
NRS6950	NRS6132b	NRS6132 *amyE::Phy-spank-mTagBFP* [19]	pNW2304 into NRS6132
NRS6951	NRS6145b	NRS6145 *amyE::Phy-spank-mTagBFP* [19]	pNW2304 into NRS6145
NRS6952	NRS6160b	NRS6160 *amyE::Phy-spank-mTagBFP* [19]	pNW2304 into NRS6160
NRS6953	NRS6181b	NRS6181 *amyE::Phy-spank-mTagBFP* [19]	pNW2304 into NRS6181
NRS6954	NRS6183b	NRS6183 *amyE::Phy-spank-mTagBFP* [19]	pNW2304 into NRS6183
NRS6955	NRS6186b	NRS6186 *amyE::Phy-spank-mTagBFP* [19]	pNW2304 into NRS6186
NRS6956	NRS6187b	NRS6187 *amyE::Phy-spank-mTagBFP* [19]	pNW2304 into NRS687
NRS6957	NRS6190b	NRS6190 *amyE::Phy-spank-mTagBFP* [19]	pNW2304 into NRS6190
NRS6958	NRS620b	NRS6202 *amyE::Phy-spank-mTagBFP* [19]	pNW2304 into NRS6202
NRS7253		168 *epeXEPAB::kan*	pNW2315 into 168
NRS7259	3610 g epe	NCIB 3610 *epeXEPAB::kan amyE::Phy-spank-gfp mut2 (cml*)	NRS7253 SPP1 into NRS6942
NRS7260	3610b epe	NCIB 3610 *epeXEPAB::kan amyE::Phy-spank-mTagBFP* [19]	NRS7253 SPP1 into NRS6932
NRS7390	NRS6153g epe	NRS6153 *epeXEPAB::kan amyE::Phy-spank-gfp mut2 (cml*)	pNW2315 into NRS6222
NRS7391	NRS6202g epe	NRS6202 *epeXEPAB::kan amyE::Phy-spank-gfp mut2 (cml*)	pNW2315 into NRS6897
NRS7392	NRS6153b epe	NRS6153 *epeXEPAB::kan amyE::Phy-spank-mTagBFP* [19]	pNW2319 into NRS6938
NRS7393	NRS6202b epe	NRS6202 *epeXEPAB::kan amyE::Phy-spank-mTagBFP* [19]	pNW2319 into NRS7201

*The naming given to strains in figures and figure legends is indicated.

†The abbreviation ‘spec’ indicates spectinomycin resistance; ‘cml’ indicates chloramphenicol resistance and ‘kan’ kanamycin resistance.

‡The method of strain construction is indicated with either the plasmid (pNW) or donor strain phage (SPP1) inserted into the parental strain.

### Strain construction

The strain used for storing of plasmids for cloning was *Escherichia coli* strain MC1061 [F’ *lacIQ lacZM15 Tn*10 [19]]. For making mutations in the NCIB 3610 background, as this strain is not genetically competent, plasmids were first transformed into the laboratory strain 168 using a standard protocol [20]. The modified region was subsequently inserted and integrated into the NCIB 3610 genome via SPP1 phage transduction [21]. For genetically competent soil isolates of *B. subtilis *, the plasmids were transformed directly into the isolate of interest as previously described [22] with the adaptations described in [14].

The *epeXEPAB* deletions in the *B. subtilis* isolates were constructed by homologous recombination and insertion of a kanamycin resistance cassette in the native locus, using plasmid pNW2315. For construction of pNW2315 the required fragment was synthesised by GenScript and inserted into the pCC1 vector. The construct sequence can be found in Table S1. Strains with the *epeXEPAB* deletion were verified by using the primers NRS2812 (5′ GTCTCGTATAATCTCTCACTTTCCC 3′) and NRS3311 (5′ AGTAAGTGGCTTTATTGATCTTGGG 3′).

For construction of the mTagBFP and GFP-expressing isolates, plasmids pNW2304 [11] and pBL165 [23] were used, respectively. Both plasmids are designed to facilitate the integration of the genes encoding the fluorescent proteins and antibiotic resistance cassettes into the *amyE* locus. Resulting colonies were therefore screened using a potato starch assay to assess loss of amylase activity [24] and expression of the appropriate fluorescent protein.

### Biofilm co-culture assays

The mixed biofilm assays were set up as previously described [11]. Cultures of the individual strains to be used were grown in 5 ml of LB at 37 °C with agitation overnight. The following morning, day cultures were set up by inoculating 3 ml of LB with 200 µl of the overnight cultures. The day cultures were incubated at 37 °C with agitation. The growth of the cultures was monitored until growth of all cultures had reached or exceeded an OD_600_ of 1, then all cultures were normalized to an OD_600_ of 1, approx. 10^6^ c.f.u. [11]. After normalization, cultures were mixed at a 1 : 1 ratio as required. Then, 5 µl drops of the culture mixtures were spotted onto MSgg agar plates and 5 µl drops of the individual normalized cultures were included in the assays as controls. The plates were incubated at 30 °C and images were taken after 24, 48 and 72 h as required. Fluorescence imaging was performed using a Leica fluorescence stereoscope (M205FCA) with a 0.5×0.2 NA objective. Imaging files were imported to OMERO [25].

### Image analysis

Relative strain densities of GFP and mTagBFP-expressing cells in mixed biofilm assays were determined by analysing fluorescent imaging data. This was done using a macro, which was kindly produced by Dr Graeme Ball at the Dundee Imaging Facility. Details of the image analysis approach follow and are as detailed in [11]: to determine relative strain densities in competitive biofilm assays using data obtained through image analysis on the total intensity of a fluorescent signal in pixels where the signal is above the background threshold. To achieve this a Fiji/ImageJ macro was written. Since images were saved as multi-series Leica LIF files this macro relies on Bio-Formats Macro Extensions to import the data. The macro can perform batch analysis of all images in a file, writing a summary table of results in CSV format as well as snapshot TIFF images showing detected biofilm regions as overlay outlines. The macro uses built-in functionality of ImageJ to detect biofilm regions, specifically: auto-thresholding using the ‘Triangle’ method after optional background subtraction using the rolling ball/sliding paraboloid algorithm. A single large colony biofilm in the centre of the image or several smaller ‘sub-colonies’ can optionally be detected (the former using the ‘Wand’ tool, the latter using ‘Analyse Particles’ with a specified size range). For the biofilm region in each image the following measurements are made for two channels: area, basic intensity statistics (mean, maximum, standard deviation, total intensity) and some colocalization statistics (Pearson's correlation coefficient, thresholded ‘object Pearsons’ and optionally Manders M1 and M2 coefficients). Finally, the percentage area within the biofilm region that is above background for each channel is reported, as well as total ‘foreground’ signal (i.e. total signal in pixels that have above-background intensity values). These final two measurements rely on a background intensity parameter for each channel that distinguishes positive expression of the label from background. For each image, the relative strain density was calculated by dividing the strain’s foreground signal by the total foreground signal in the image. Graphs were constructed using GraphPad prism 7.

### Enhanced whole-genome sequencing

Enhanced whole-genome sequencing was performed by MicrobesNG. This required a combination of Illumina short-read data acquisition and nanopore sequencing for long-read data. For the preparation of samples, a single colony of each strain to be sequenced was resuspended in 200 µl of sterile PBS buffer and 100 µl of this was used to inoculate 300 ml of LB broth. The remaining 100 µl was streaked on an LB agar plate, which was incubated at 37 °C overnight. The 300 ml culture was incubated at 16 °C with shaking overnight. The following morning, the culture was incubated at 37 °C with shaking and the OD_600_ was monitored. When cultures had reached an OD_600_ value of between 0.5 and 0.8, they were centrifuged at 3750 r.p.m. for 10 min. The supernatant was removed, and the pellets were resuspended in a tube with a cryopreservative (Microbank, Pro-Lab Diagnostics UK, United Kingdom) or with DNA/RNA Shield (Zymo Research, USA) following MicrobesNG strain submission procedures. The weight of the pellet required for *B. subtilis* submission was at least 1 g, so all samples were grown in large enough volumes to exceed 1 g of pelleted cells. The spread plate set up at the same time as the culture was used for quality assessment, to ensure no contamination had occurred. The samples were sent to the MicrobesNG facilities. There, for DNA extraction, 5 to 45 µl of the suspension was lysed with 120 µl of TE buffer containing lysozyme (final concentration 0.1 mg ml^−1^) and RNase A (ITW Reagents, Barcelona, Spain) (final concentration 0.1 mg ml^−1^), incubated for 25 min at 37 °C. Proteinase K (VWR Chemicals, Ohio, USA) (final concentration 0.1 mg ml^−1^) and SDS (Sigma-Aldrich, Missouri, USA) (final concentration 0.5 % v/v) were added and incubated for 5 min at 65 °C. Genomic DNA was purified using an equal volume of SPRI beads and resuspended in EB buffer (Qiagen, Germany). DNA was quantified with the Quant-iT dsDNA HS kit (ThermoFisher Scientific) assay in an Eppendorf AF2200 plate reader (Eppendorf UK, United Kingdom). For Illumina sequencing, genomic DNA libraries were prepared using the Nextera XT Library Prep Kit (Illumina, San Diego, USA) following the manufacturer’s protocol with the following modifications: input DNA was increased twofold, and PCR elongation time was increased to 45 s. DNA quantification and library preparation were carried out on a Hamilton Microlab STAR automated liquid handling system (Hamilton Bonaduz AG, Switzerland). Pooled libraries were quantified using the Kapa Biosystems Library Quantification Kit for Illumina. Libraries were sequenced using Illumina sequencers (HiSeq/NovaSeq) using a 250 bp paired end protocol. Long-read genomic DNA libraries were prepared with Oxford Nanopore SQK-RBK004 kit and/or SQK-LSK109 kit with Native Barcoding EXP-NBD104/114 (ONT, United Kingdom) using 400–500 ng of HMW DNA. Barcoded samples were pooled together into a single sequencing library and loaded in a FLO-MIN106 (R.9.4.1) flow cell in a GridION (ONT, United Kingdom).

### Genome assembly

Illumina reads were adapter trimmed using Trimmomatic 0.30 with a sliding window quality cutoff of Q15 [26]. An initial nanopore-only genome assembly was carried out using Flye 2.9.1 [27] with the ‘nano-raw’ model, and the resulting contigs used in conjunction with the Illumina reads with Unicycler v0.5.0 [28] using ‘bold’ mode to produce a final assembly. The resulting contigs were annotated using bakta 1.40 (database version 3.1) [29]. Examination of the assembly graphs allowed putative plasmid sequences to be identified in cases where short, circular molecules were evident, which were not integrated into the chromosomal sequence. Raw sequence reads and annotated assemblies can be found under European Nucleotide Archive Project PRJEB43128.

### Phylogenetic tree construction

The nucleotide sequences of *gyrA*, *rpoB*, *dnaJ* and *recA* were extracted from the short-read data (which can be found in our previous publication [14]) using Artemis [30] and concatenated. The same sequences for the reference strain *B. subtilis* NCIB 3610 (GenBank accession number GCA_002055965.1) were retrieved from NCBI, concatenated and included in the analysis. The sequences were aligned in Jalview [31] by mafft using the G-INS-I algorithm and mega7 software [32] was used to construct a maximum-likelihood phylogenetic tree with 100 bootstrap repeats as previously described [14].

### Pangenome analysis

A pangenome analysis of all environmental isolates included in this work, the model isolate NCIB 3610 and other publicly available genome sequences of *B. subtilis* isolates was constructed using Roary version 3.13.0 with default parameters. The draft genome assemblies were used as the input. The pangenome figure was produced using the roary_plots.py macro and further annotated in Adobe Illustrator (https://adobe.com/products/illustrator).

### Command line blast

To explore the presence and distribution of the genes within the *epeXEPAB* cluster, command line blast was used to create a nucleotide database using the whole genomes of NCIB 3610 and the 21 genetically competent isolates in our collection. The database was then used to perform nucleotide blast searches of the *epe* genes. The outcome of the analysis and locations of genes of interest were used to manually extract the sequences of interest. The sequences were aligned and exported as image files in Jalview [31] to explore the diversity in the coding sequences where required.

### antiSMASH

To determine the secondary metabolite biosynthesis clusters encoded by each isolate, antiSMASH version 6.0 was used [33]. Enhanced whole-genome sequence assemblies were submitted to the server and run with default settings. GenBank files of all secondary metabolite biosynthesis clusters encoded by all isolates were retained.

### Clinker

Clinker version 0.0.20 was used with default settings to visualize the secondary metabolite biosynthesis clusters identified by antiSMASH. The GenBank files of the clusters downloaded from antiSMASH were used as an input for clinker to produce figures. The figures were modified using Adobe Illustrator (https://adobe.com/products/illustrator).

## Results

### Mixed biofilm intra-species competition and phylogenetic relatedness

**Fig. 1.**
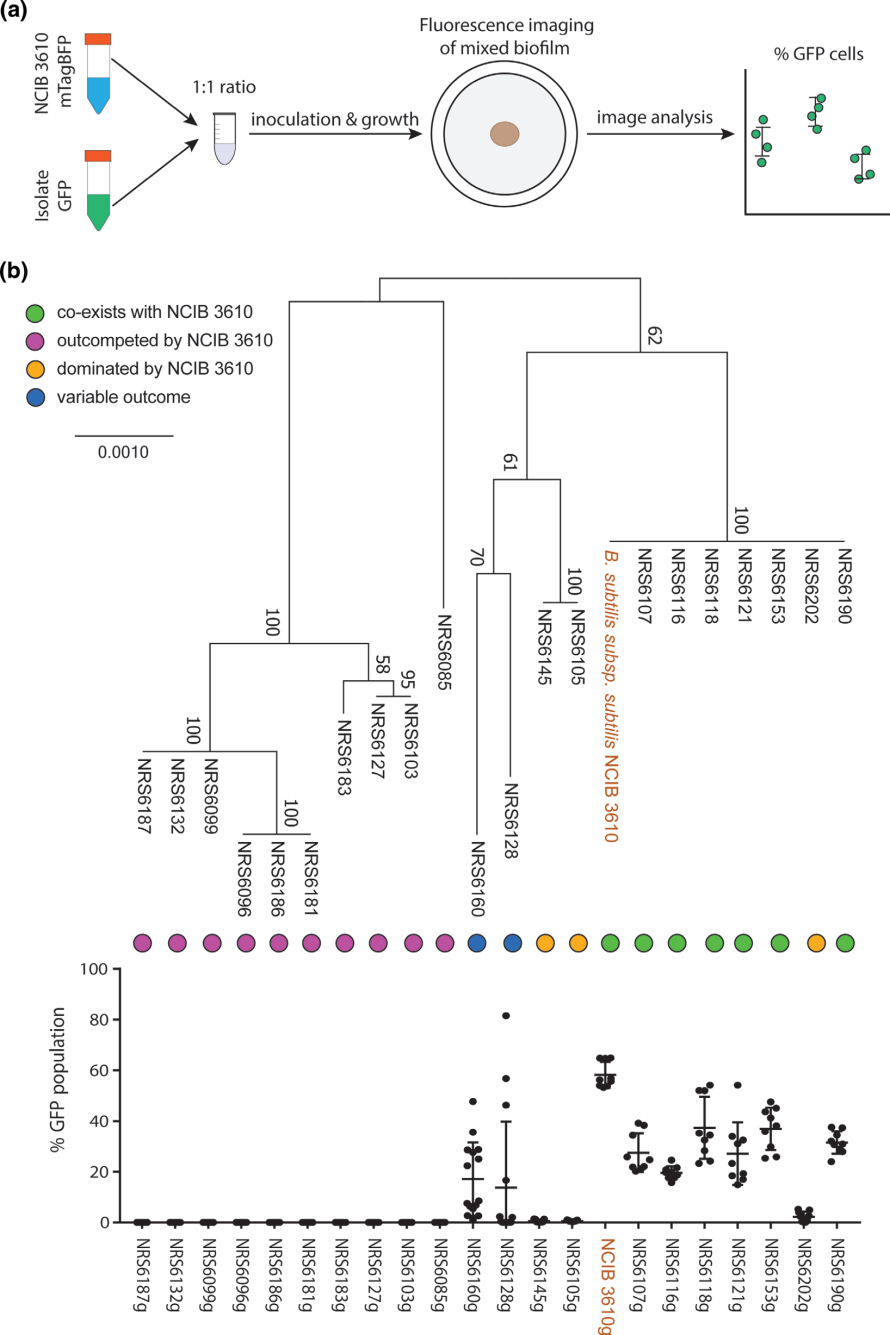
Mixed biofilm intra-species competition outcomes of *B. subtilis* isolates against the model NCIB 3610. (**a**) Schematic representation of mixed biofilm setup. (**b**) Maximum-likelihood phylogenetic tree based on the concatenated sequences of housekeeping genes *gyrA*, *rpoB*, *dnaJ*, *recA* shown alongside the competition outcomes of mixed biofilms of NCIB 3610 co-incubated with each of the 21 environmental isolates of *B. subtilis* used in this work for 24 h. The presented values are the % of the community of GFP expressing soil isolates, quantified using image analysis. The nine data points presented for each isolate represent three biological repeats and three technical repeats. The error bars represent the standard deviation of the mean.

We examined the competitive outcome of the interaction between 21 *B. subtilis* soil isolates and the model isolate NCIB 3610 in the context of a mixed isolate colony biofilm. The library of undomesticated *B. subtilis* isolates were collected as part of a previous study [14]. We used isolates that demonstrated at least a low frequency of genetic competency due to the necessity to be able to distinguish the isolates in assays. In each competition interaction, we competed a variant of NCIB 3610 that constitutively expresses mTagBFP against the GFP expressing variants of the soil isolates. We also included an NCIB 3610 isogenic mix as a control. Each colony biofilm was founded with approx. 10^6^ c.f.u. [11]. We imaged the colony biofilms after 24, 48 and 72 h of incubation at 30 °C (Fig. S1, available in the online version of this article). We quantified the proportion of GFP-expressing cells in the mixed biofilm using our previously established and validated image analysis method [11] (Fig. 1a). It is relevant to note that we have previously shown that the percentage of the colony biofilm occupied by each strain and quantified using the image analysis approach directly correlates with data obtained by flow cytometry [11]. Analysis of the NCIB 3610 isogenic control revealed that the GFP variant typically comprises approximately 60 % of the community. As a 1 : 1 ratio between GFP and mTagBFP variants of NCIB 3610 is expected, the slight under representation of the mTagBFP variant is perhaps due to differences in fitness associated with the different fluorescent proteins (Fig. 1b). The underrepresentation of the strain carrying mTagBFP is consistent with our previous observations and did not preclude us from defining the relationships between the isolates [11].

The outcome of competition between the pairs of isolates shows that NCIB 3610 is a strong competitor that outcompetes most soil isolates from the 24 h time point (Figs 1b and S1). It is also evident that, for some isolate pairs where co-existence is observed at the 24 h timepoint, the proportion of soil isolate in the community decreases over time, for example, see NRS6190 (Figs. S1, S2). Based on the outcome of their interaction with NCIB 3610 after 24 h of co-incubation, we defined the isolates in our collection as ‘outcompeted’ (those that took up 0 % of the community), ‘dominated’ (those that took up 0–5 % of the community), ‘co-existing’ (those that took up more than 5%) and ‘variable’ (those that in some rounds were dominated and in others co-existed) (Figs 1b and S2A) using these custom thresholds.

*B. subtilis* intra-species interactions have primarily been studied in the context of kin discrimination, which is defined as the differential treatment of conspecific isolates based on phylogenetic relationship [9, 34–38]. Therefore, we correlated the outcome of the mixed biofilm screens with a maximum-likelihood tree based on the concatenated nucleotide sequences of four housekeeping genes (*gyrA*, *rpoB*, *recA*, *dnaJ*). Our results show there is a correlation between the ability of isolates to co-exist with NCIB 3610 and how related the isolates are. All isolates that co-exist with NCIB 3610 are in the same phylogenetic group. Only one isolate of this group (namely NRS6202) fell within the class of isolates that were dominated by NCIB3610. The remaining two isolates that were in the ‘dominated’ group, along with the two isolates that show ‘variable’ results, are more distantly related to NCIB 3610. All isolates that are ‘outcompeted’ by NCIB 3610 form the most distantly related phylogenetic groups (Fig. 1b). This analysis indicates that the outcome of the interactions between our isolates are broadly consistent with the concept of kin discrimination.

#### Pangenome analysis of soil isolates of *B. subtilis*

**Fig. 2.**
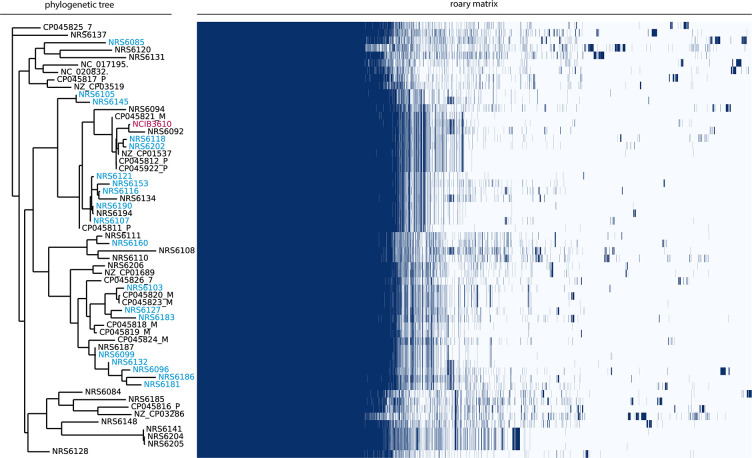
Pangenome analysis and phylogeny of *B. subtilis* isolates. The names of genetically competent soil isolates from the NSW laboratory are coloured in blue on the phylogenetic tree shown on the left. Non-competent isolates in the NSW lab collection and publicly accessible genomes from diverse sources are coloured in black. The model isolate NCIB 3610 is shown in pink. The Roary matrix shows the presence (blue) and absence [19] of genes in each isolate.

The 21 isolates of *B. subtilis* used in this work have been isolated from soil samples in Scotland [14]. To explore the genomic diversity of these isolates, we used short-read sequence data [14] and performed a pangenome analysis using Roary [39]. We included all the isolates in our collection [14] alongside other randomly selected publicly accessible closed genome sequences intended to provide coverages of other geographic locations and isolation sources. The analysis shows that there is a large diversity in the accessory genes found within the isolates examined. Additionally, the phylogenetic distribution of the isolates in our collection is varied, with isolates positioned within different clades (Fig. 2). Importantly, the analysis shows that the isolates in our collection, while sampled locally, provide a good representation of the diversity found among more widely sampled *B. subtilis* isolates. To facilitate further bioinformatic analysis we acquired the enhanced whole-genome sequences for the isolates (MicrobesNG, Birmingham, United Kingdom). After receiving the Illumina reads and long-read data, the genomes were quality assessed and re-assembled to incorporate our initial Illumina data [14] and consequently increase coverage (Table S2) (ENA Project PRJEB43128).

### Exploring the specialized metabolite biosynthesis clusters encoded by the isolates in our collection

**Fig. 3.**
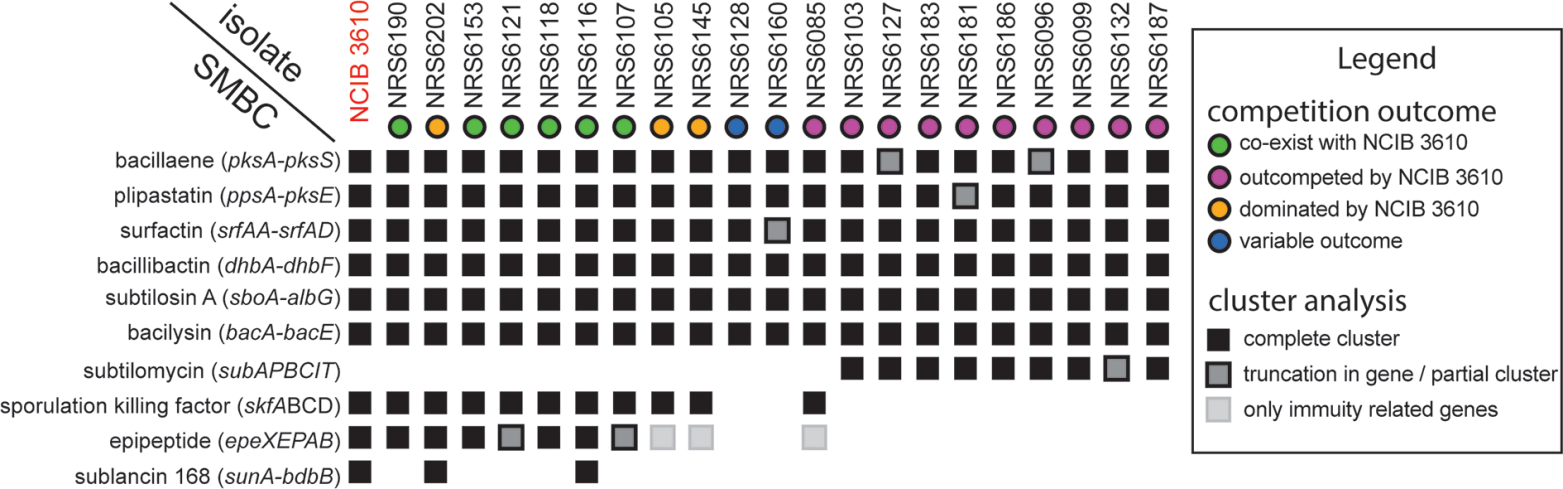
Secondary metabolite biosynthesis clusters and competitive fitness of soil isolates of *B. subtilis *. The specialized metabolites on the left-hand side represent the molecules encoded by each cluster identified by antiSMASH [33]. The NCIB 3610 and ‘NRS’ followed by numbers at the top represent different isolates used in this study. The outcomes of competitions in biofilms are indicated by coloured circles. This data is presented in Fig. 1(b) and are as shown in the legend. The coloured squares show the presence and any variations in the encoded clusters and what they represent is shown in the legend.

To uncover the specialized metabolite biosynthesis clusters (SMBC) encoded by each of the isolates in our collection we used antiSMASH version 6.0 [33], a tool designed for mining bacterial genomes and detecting such clusters. We correlated the presence of SMBCs that have a known antimicrobial function with the competitive phenotype of our isolates (Fig. 3). In some cases, sequence variations and truncations were found in SMBCs for a small subset of isolates (Figs 3 and S3). The core clusters, a version of which was present in all isolates in our collection, are those required for the biosynthesis of bacillaene [40], plipastatin [41], bacillibactin [42], surfactin [43], subtilosin A [44] and bacilysin [45]. One hypothesis is that the differential regulation of the core clusters could explain the competition outcome. However, here we focused on clusters that were not contained in all the genomes, which produce metabolites with known antimicrobial properties, as we considered these likely to be involved in intra-species competition. The variable clusters encoded in our collection of *B. subtilis* isolates were those responsible for producing subtilomycin [46], sporulation killing factor [47–49], epipeptide [15, 18] and sublancin 168 [50, 51] (Fig. 3, Table S3). The cluster, which most closely correlated with competitive fitness, was the operon encoding for the epipeptide EpeX, as only NCIB 3610 and isolates that could survive in a biofilm in the presence of NCIB 3610 encoded either the entire cluster or at least the immunity related genes (Fig. 3). As the epipeptide EpeX is a relatively newly discovered molecule and little is known about its impact in biofilm settings, we chose to further investigate the potential role of EpeX in shaping competitive interactions in mixed biofilms.

### EpeX is a potential competition determinant

**Fig. 4.**
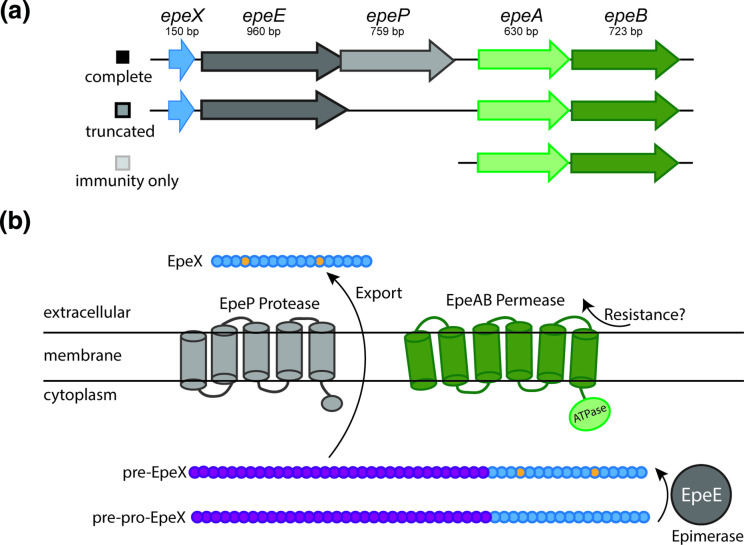
EpeX as a potential competition determinant of intra-species interactions. (**a**) Schematic representation of the variants of the *epeXEPAB* found in the genomes of the isolates used in this work. The coloured boxes next to each cluster schematic identify the cluster variant; (**b**) schematic representation of the components and function of EpeXEPAB. Amino acids coloured in yellow for the pre-EpeX indicate amino acids epimerised by EpeE prior to being cleaved and is presumably further processed and exported from the cell. The processing and export are thought to be mediated by the EpeP protease to generate the final form, EpeX. The EpeAB permease is believed to be involved in immunity against EpeX. The schematic has been adapted from [18].

The *epe* cluster of *B. subtilis* NCIB 3610 consists of *epeX, epeE, epeP, epeA* and *epeB* (Fig. 4a). The variants of the cluster found within our isolate collection are presented (Fig. 4a) and full details are provided (Table S4, Figs. S4, S5, S6). EpeX has a toxic effect on the cell envelope of *B. subtilis* [15, 16]. It is made as pre-pro-peptide in the cytoplasm that is processed by the radical-S-adenosyl-l-methionine [19] epimerase EpeE, which converts the l-valine and l-isoleucine of EpeX into their d-configured counterparts generating pre-EpeX [17]. Pre-EpeX is further exported and cleaved, and based on the genomic arrangement, it is predicted that this is mediated by EpeP, a membrane anchored signal peptidase [18]. Finally, EpeAB form an ABC transporter that confers partial resistance to the intrinsically produced EpeX and is involved in autoimmunity [16] (Fig. 4b). The EpeX peptide triggers the activation of the LiaRS-dependent cell-envelope stress response, and LiaH (phage heat-shock protein) and LiaI (membrane anchor) are additional major resistance determinants against the antimicrobial peptide. Consistent with the cell-envelope stress reponse being involved in immunity against the epipeptide, the mode of action of EpeX is membrane depolarization, which causes permeabilization of the membrane [15]. This makes EpeX a likely candidate for a role in intra-species interactions and kin discrimination.

### Absence of the *epeXEPAB* cluster impacts competition against an otherwise isogenic strain

**Fig. 5.**
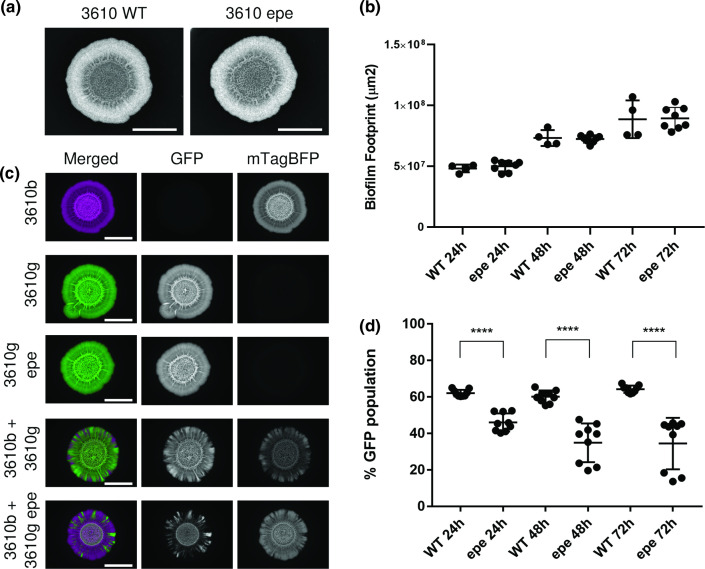
Competition assay outcome between NCIB 3610 wild type and *epeXEPAB* mutants. (**a**) Representative images of single strain biofilms of the wild-type (‘WT’) and *epeXEPAB* (‘epe’) mutant of NCIB 3610 (‘3610’) grown on MSgg media for 48 h at 30 °C. The scale bars represent 0.5 cm. (**b**) The area occupied by the colony biofilms was quantified ‘WT’ for the wild-type NCIB 3610 and ‘epe’ is the *epeXEPAB* deletion mutant of NCIB 3610. (**c**) Representative images of biofilms growth for 48 h at 30 °C on MSgg agar. ‘3610’ is the model isolate NCIB 3610. Strain names followed by ‘b’ represent strains constitutively expressing mTagBFP, false coloured in magenta and names followed by ‘g’ represent strains constitutively expressing GFP and are false coloured in green. ‘epe’ represents deletion of the *epeXEPAB* operon. ‘3610b’ and ‘3610 g epe’ are images of the same biofilms as those shown in (**a**). The scale bars represent 0.5 cm. (**d**) Competition results of NCIB 3610 wild-type (‘WT’) expressing mTagBFP (NRS6932) against GFP-expressing wild type (NRS6942) or *epeXEPAB* mutant (NRS7259) of NCIB 3610 (‘epe’) as indicated after 24, 48 and 72 h of co-incubation on biofilm-inducing media plates. The presented values are the % of the community of GFP expressing strain, quantified using image analysis. Each individual data point presented for each isolate represent one of two or three technical replicates for the three biological repeats performed. The error bars represent the standard deviation of the mean. The asterisks represent statistical significance with a *P* value of ≤0.0001 between the two populations as calculated using an unpaired *t*-test.

To investigate if the *epeXEPAB* cluster has a role in shaping intra-species interactions in the context of a mixed isolate colony biofilm, we constructed a variant of NCIB 3610 that lacks the entire *epeXEPAB* cluster. We tested the competitiveness of this mutant against NCIB 3610 in mixed isolate colony biofilms. From the single isolate controls, it is apparent that, at least on a macroscopic level, colony morphology is not impacted by the absence of the *epeXEPAB* cluster (Fig. 5a). Moreover, the area occupied by the colony biofilms formed by each strain were the same (Fig. 5b), suggesting no major impact of deleting the *epeXEPAB* cluster on growth. To determine the outcome of the competition between the strains in the mixed colony biofilms, we again used image analysis to quantify the proportion of GFP and mTagBFP expressing cells in the community that developed. Our results show that the *epeXEPAB* mutant of NCIB 3610 is less successful than the wild-type, as the proportion of the community it occupied is significantly lower than that taken up by the wild-type in the isogenic control sample (Fig. 5c, d). These data show that the *epeXEPAB* cluster is a determinant of the competition outcome in an otherwise isogenic biofilm co-culture. Lack of this cluster decreases the competitive strength of *B. subtills* NCIB 3610.

### A limited role for EpeX as an intra-species competition determinant

**Fig. 6.**
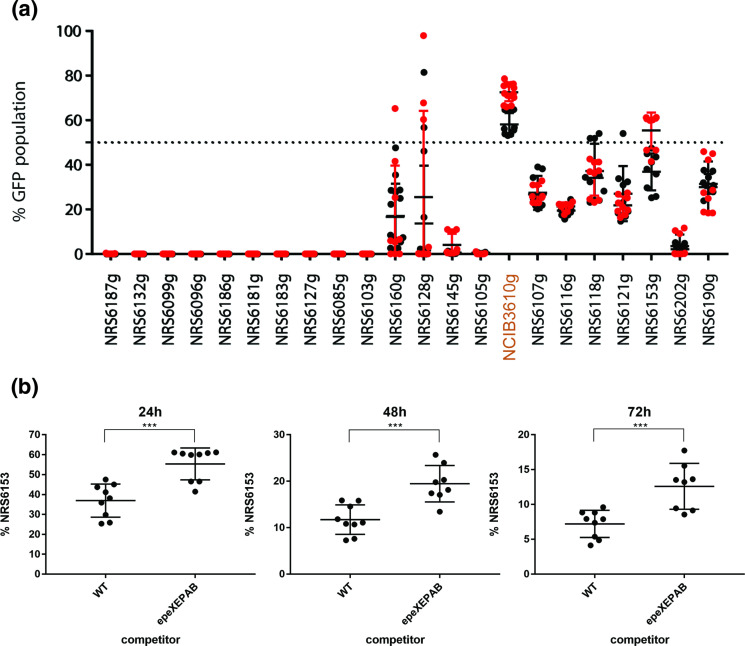
Comparison of mixed biofilm outcomes using NCIB 3610 wild-type and *epeXEPAB* as references. (**a**) Competition results of NCIB 3610 wild type (WT) expressing mTagBFP (NRS6932, black data points) or NCIB 3610 *epeXEPAB* expressing mTagBFP (NRS7260, red data points) against GFP-expressing soil isolates at 24 h of co-incubation on biofilm inducing media plates as indicated. The presented values are the % of the community of GFP expressing soil isolates, quantified using image analysis. Each individual data point presented for each isolate represent one of two or three technical replicates for three biological repeats with each reference strain as indicated. The error bars represent the standard deviation of the mean. (**b**) Competition results of GFP-expressing NRS6153 (NRS6222) against *mTagBFP*-expressing wild-type (NRS6932) or *epeXEPAB* mutants (NRS7260) of NCIB 3610 after 24, 48 and 72 h of co-incubation on biofilm inducing media plates as indicated. The presented values are the % of the community of GFP expressing strain (NRS6153), quantified using image analysis. Each individual data point presented for each isolate represent one of two or three technical replicates for the three biological repeats performed. The error bars represent the standard deviation of the mean. The asterisks represent statistical significance with a *P* value of ≤0.001 between the two populations as calculated using an unpaired *t*-test.

Next, we explored how NCIB 3610 lacking the *epeXEPAB* cluster competed when mixed with the 21 soil isolates in our collection. We hypothesized that if EpeX is a competition determinant of intra-species interactions, then the lack of *epeXEPAB* would reduce the competitive fitness of NCIB 3610. This would allow for (a) underrepresentation of the NCIB 3610 *epeXEPAB* strain in cases where co-existence was achieved with the wild-type, and/or (b) isolates that are outcompeted or dominated by the wild NCIB 3610 managing to achieve some level of co-existence with the *epeXEPAB* mutant. We used an mTagBFP-expressing variant of NCIB 3610 *epeXEPAB* as a reference strain, competing it against our suite of GFP-expressing isolates, and overlayed the data from this screen with the data obtained from the screen of all isolates against the wild-type NCIB 3610 (recall Fig. 1b). Our results show that for most of the isolates, the loss of the *epeXEPAB* cluster in NCIB 3610 has no impact on the outcome of the pairwise competition (Fig. 6a). The only isolate that takes up a larger portion of the community when mixed with the *epeXEPAB* mutant versus the wild-type of NCIB 3610 is isolate NRS6153. To explore this relationship more closely, we further analysed the data and found that there is a statistically significant difference between the portion of the community taken up by NRS6153 when mixed with the two variants of NCIB 3610 (Fig. 6b). However, deletion of the *epeXEPAB* cluster in NRS6153 did not impact competition with its otherwise isogenic parental strain (Fig. S7A). This was also the case for isolate NRS6202 (Fig. S7B), another isolate in the collection that carries the complete *epeXEPAB* operon. Collectively, our data uncover a limited role for EpeX as a competition determinant of *B. subtilis* intra-species interactions but reveal that the impact that EpeX has varies greatly depending on the competing isolate.

## Discussion

In this study, we combined bioinformatic analysis and co-culture experiments to identify a new competition determinant of *B. subtilis* intra-species interactions that is active within a spatially confined colony biofilm. We first assessed the outcomes of pair-wise competitions of 21 soil isolates challenged against NCIB 3610 in a mixed biofilm and found that NCIB 3610 is a strong competitor, which outcompeted or dominated the majority of environmental isolates used in this work. Interestingly, no isolates were found that could outcompete or dominate NCIB 3610. In fact, even the minority of isolates that were able to co-exist with NCIB 3610, consistently took up less than 50 % of the community by the 48 h timepoint. We speculate that the strong competitive nature of NCIB 3610 could be a result of laboratory adaptation. Indeed, NCIB 3610 has previously been reported to be an ‘atypical’ isolate in terms of its social behaviours. This is due to a mutation affecting its quorum sensing, which is believed to be the result of laboratory domestication [52, 53]. This atypical signalling has been shown to impact a plethora of behaviours such as biofilm formation [53], growth [52] and expression of some specialised metabolites [54] and is therefore likely to also impact competitive fitness in a laboratory setting. By examining the outcome of the competition assays alongside the genome data of each of the isolates, we found a correlation between isolates encoding the cluster responsible for producing the epipeptide EpeX and competitive fitness. The *epeXEPAB* cluster is widely distributed among firmicutes and presence of the EpeX peptide causes cell-envelope stress response and cell death via membrane perturbation in *B. subtilis* [15, 17, 18]. We therefore hypothesized that this cluster was (in part) responsible for increasing the competitive fitness of isolates in a conspecific competition setting. To test our hypothesis, we deleted this cluster in NCIB 3610 and performed competitions of the mutant against both the wild-type NCIB 3610 and our collection of soil isolates. We found that lack of the cluster responsible for EpeX production led to a decrease in competitive fitness in an otherwise isogenic context for NCIB 3610. When the variant of NCIB 3610 lacking the *epe* cluster was competed against the rest of the isolates in the strain collection it displayed the same competitive strength as the parental isolate for 20 of the 21 isolates. The exception was isolate NRS6153, which occupied a significantly larger portion of the mature colony biofilm community when mixed against the *epeXEPAB* mutant compared with its pairing with the wild-type NCIB 3610. Additionally, looking beyond the model isolate NCIB 3610, when we deleted the *epeXEPAB* cluster in isolates NRS6153 and NRS6202, no impact on competitive fitness was observed. We cannot preclude that a bias in the competition outcome has been introduced by our use of only isolates of *B. subtilis* that demonstrated at least a low frequency of genetic competency. The selection of strains with this characteristic was unavoidable given the experimental approach.

The identification of EpeX as a competition determinant within the spatially confined colony biofilm indicates that the epipeptide must be produced in these conditions. If the production of EpeX did not coincide with the conditions used, no impact of deleting the EpeX gene cluster would be observed. Activity of the epipeptide within a colony biofilm is consistent with what is known about the expression profile of the *epe* operon. A critical regulator of biofilm matrix production and sporulation, Spo0A [55] relieves the repression of *epe* transcription via AbrB to allow EpeX to be produced [18]. The reason why there is an isolate-specific response to the presence of the epipeptide between different isolates remains to be explored. One possible explanation is the fact that immunity against EpeX is complex and is largely achieved through activation of the broad cell-envelope stress response, orchestrated by the LiaRS two-component system [15, 16]. Therefore, potential differences in the timing and combination of cell-wall targeting competition determinants under the conditions tested could result in various levels of susceptibility of target cells to EpeX and the observed differences in the impact that this molecule has on competition. One way to explore how NCIB 3610 induces LiaRS response in different isolates could be using transcriptional reporter fusions with the promoter of the LiaRS system in both isolates that are impacted by EpeX and those that are not.

### Overarching conclusion

Specialized metabolites are important determinants of social interactions among bacteria. While it is known that some specialized metabolites impact kin discrimination in the context of swarm meeting assays [9], it was unknown if and how different specialized metabolites affect the competitive strength of an isolate against conspecific isolates in a mixed biofilm. As biofilm formation is a very different physiological state to swarming [56] it is unknown if the molecules that affect mixing of swarms will be the same as those impacting competition in a biofilm setting. Additionally, the swarm meeting assays used previously to define the molecular determinants of kin discrimination [9] do not give any information about the competitive fitness of individual isolates, but rather just determine whether two strains can share a niche or not. In this work we addressed some of these knowledge gaps and revealed EpeX to be a novel competition determinant among some *B. subtilis* isolates.

## Supplementary Data

Supplementary material 1
