# Supplementary material for: The putative role of the epipeptide EpeX in Bacillus subtilis intra-species competition
Source: Microbiology (Reading). 2023 Jun 8;169(6):001344. doi: 10.1099/mic.0.001344 (PMC7614699; doi:10.1099/mic.0.001344)
Supplement: Supplementary material 1 [file mic-169-1344-s001.pdf]

Supplemental Material

The putative role of the epipeptide EpeX in *Bacillus subtilis* intra-species competition

Margarita Kalamara[1], James Abbott[2], Tetyana Sukhodub[1], Cait MacPhee[3], Nicola. R. Stanley-Wall[1*]

[1] Division of Molecular Microbiology, School of Life Sciences, University of Dundee, Dundee, DD5 4EH, UK

[2] Data Analysis Group, Division of Computational Biology, School of Life Sciences, University of Dundee, Dundee, DD5 4EH, UK

[3] National Biofilms Innovation Centre, School of Physics & Astronomy, University of Edinburgh, EH9 3FD Edinburgh, UK

*** For contact:**

Prof Nicola Stanley-Wall n.r.stanleywall@dundee.ac.uk

Keywords: *Bacillus subtilis*, biofilm, kin discrimination, epipeptide, EpeX

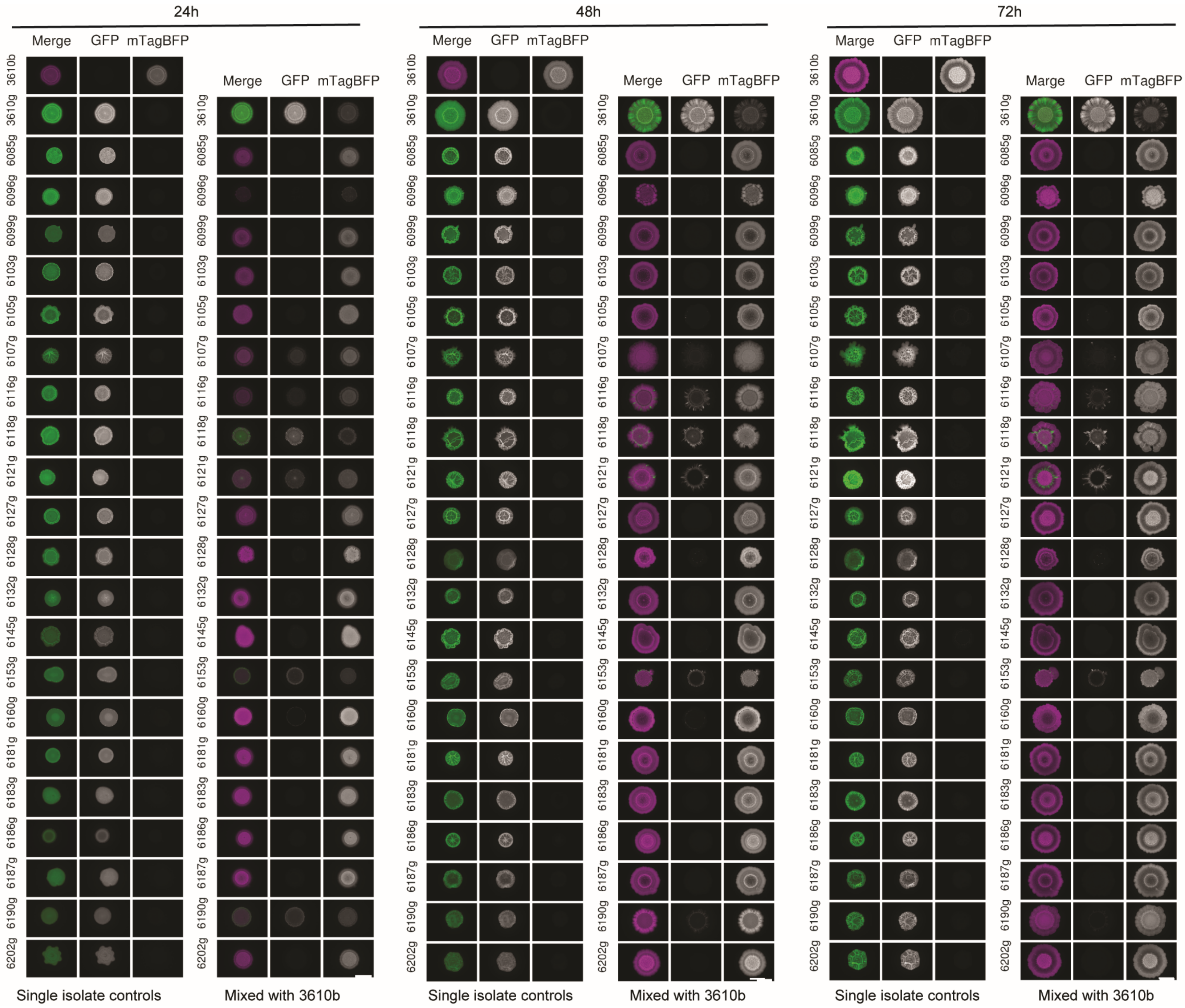

24h
48h
72h
Merge
GFP
mTagBFP
Marge
3610b
3610g
6085g
6096g
6099g
6103g
6105g
6107g
6116g
6118g
6121g
6127g
6128g
6132g
6145g
6153g
6160g
6181g
6183g
6186g
6187g
6190g
6202g
Single isolate controls
Mixed with 3610b

Figure S1: Mixed isolate biofilm screen with NCIB 3610 as a reference. Representative images from the mixed biofilm screen. Single isolate controls and mixed biofilms using NCIB 3610 mTagBFP as a reference are shown, taken after 24, 48 and 72h of growth as indicated. 3610 represents the model isolate NCIB 3610 and other numbers represent NRS numbers given to soil isolates in our collection (See Table 1). Isolate names followed by “g” represent strains constitutively expressing GFP, false coloured in green here, and isolate names followed by “b” represent strains expressing mTagBFP, false coloured in magenta. The labels at the top of each vertical panel represent the fluorescent channel images shown where “merge” shows both the GFP and mTagBFP channels. The scale bars represent 0.5 cm.

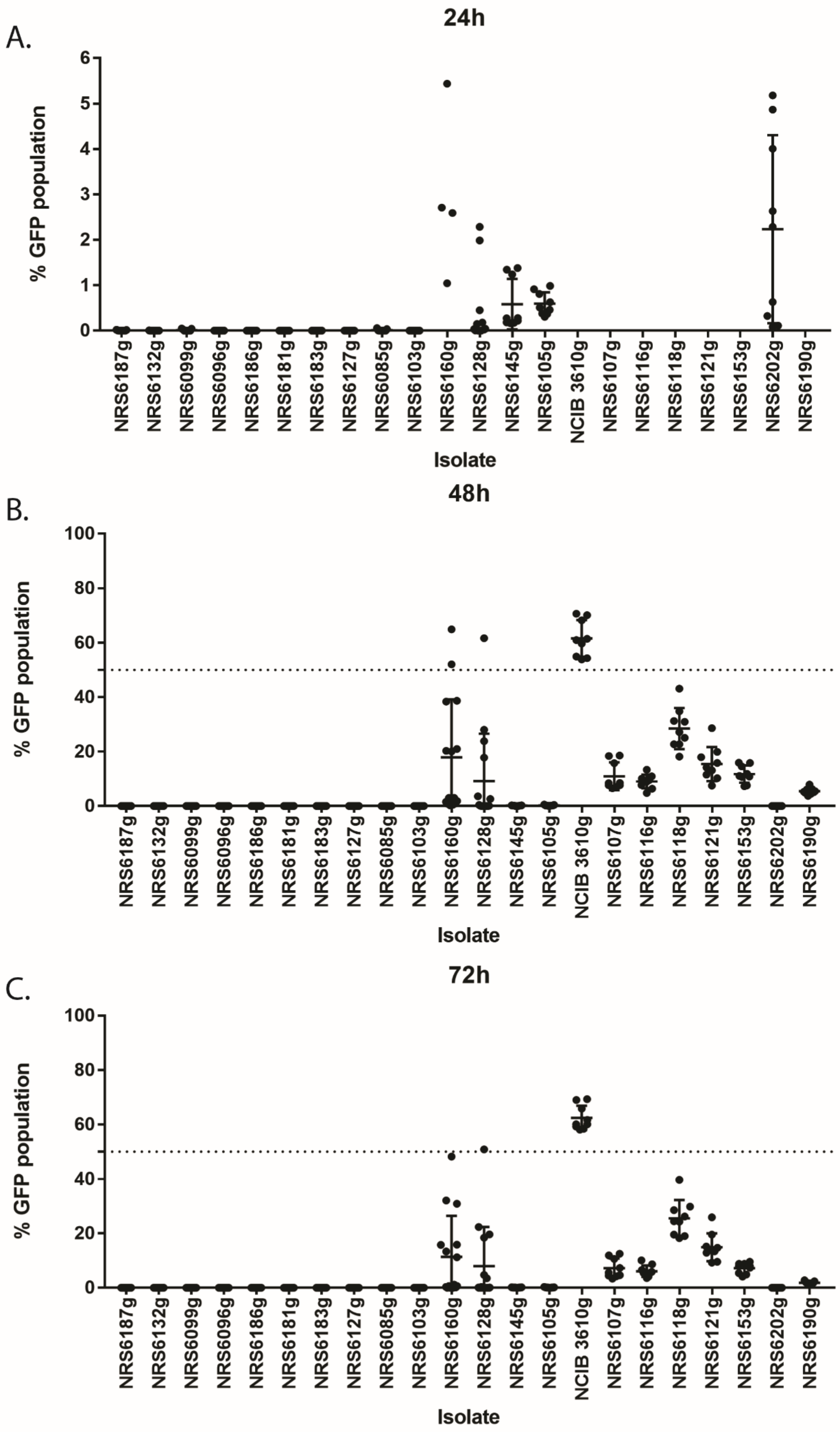

A.
24h
% GFP population
0
1
2
3
4
5
6
NRS6187g
NRS6132g
NRS6099g
NRS6096g
NRS6186g
NRS6181g
NRS6183g
NRS6127g
NRS6085g
NRS6103g
NRS6160g
NRS6128g
NRS6145g
NRS6105g
NCIB 3610g
NRS6107g
NRS6116g
NRS6118g
NRS6121g
NRS6153g
NRS6202g
NRS6190g
Isolate
B.
48h
% GFP population
0
20
40
60
80
100
NRS6187g
NRS6132g
NRS6099g
NRS6096g
NRS6186g
NRS6181g
NRS6183g
NRS6127g
NRS6085g
NRS6103g
NRS6160g
NRS6128g
NRS6145g
NRS6105g
NCIB 3610g
NRS6107g
NRS6116g
NRS6118g
NRS6121g
NRS6153g
NRS6202g
NRS6190g
Isolate
C.
72h
% GFP population
0
20
40
60
80
100
NRS6187g
NRS6132g
NRS6099g
NRS6096g
NRS6186g
NRS6181g
NRS6183g
NRS6127g
NRS6085g
NRS6103g
NRS6160g
NRS6128g
NRS6145g
NRS6105g
NCIB 3610g
NRS6107g
NRS6116g
NRS6118g
NRS6121g
NRS6153g
NRS6202g
NRS6190g
Isolate

Figure S2: Outcome of biofilm co-culture screen with NCIB 3610 as a reference. Competition results of NCIB 3610 mTagBFP against GFP-expressing soil isolates at 24 (A), 48 (B), and 72 (C) hours of co-incubation on biofilm-inducing media plates. The presented values are the % of the community of GFP expressing soil isolates, quantified using image analysis. The nine data points presented for each isolate represent three biological repeats and three technical repeats. The error bars represent the standard deviation of the mean. Note that data points in (A) are shown on a smaller scale for clarity and the full dataset is shown in Figure 1B. The dotted line in B and C represents the outcome if both strains were at equal levels in the final community. The raw data used for plotting is provided in Supplemental Appendix 2.

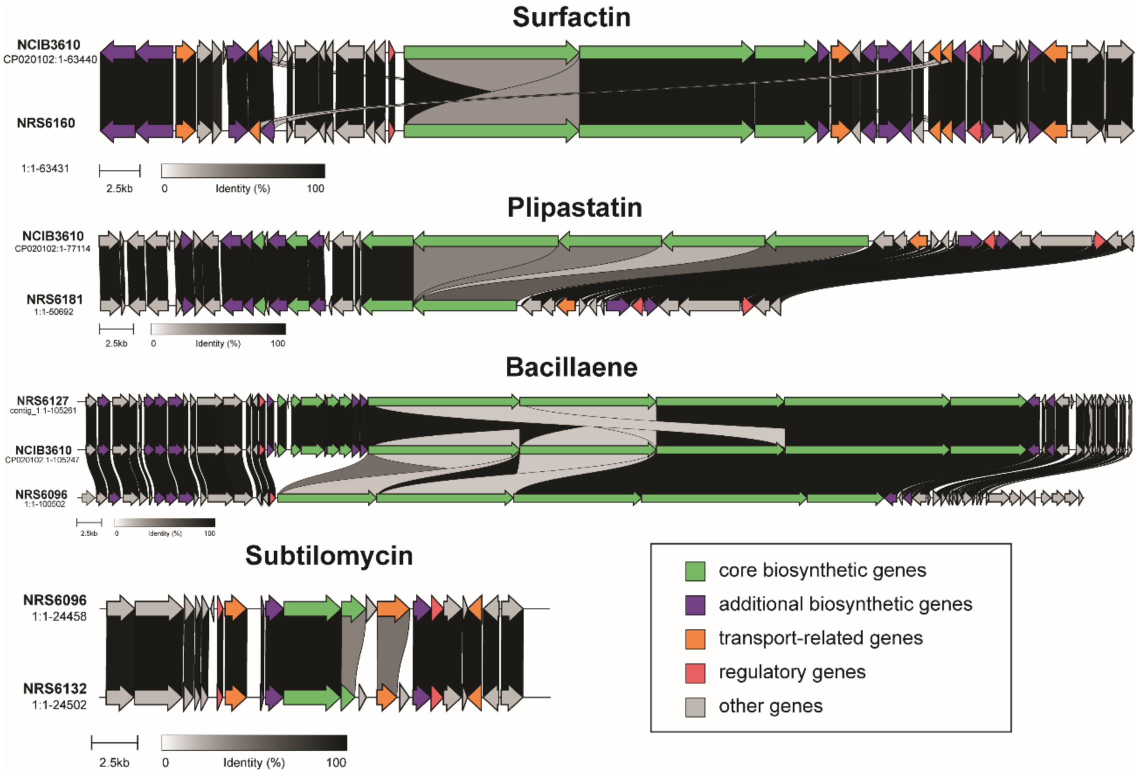

Figure S3: Schematic representation of clusters found in *B. subtilis* genomes. Shown are clusters that have some level of sequence variability from the consensus sequence of each of these clusters found within our isolate collection. In all cases, an isolate showing the default cluster is at the top with the variant cluster(s) found at the bottom. The diagrams were generated using clinker (1), using gbk files of the clusters produced by antiSMASH 6.0 (2) as input. The colour-coding annotation of the genes is described in the legend in the bottom right corner. The annotations presented here are based on the predicted function of each of these genes by antiSMASH.

A.

1 M K I L K Y E D E K Y E V L V Q N N V F
1 ATGAAAATTTTAAAGTATGAAGATGAAAAATATGAAGTGCTTGTGCAAAACAACGTATTT

21 I K D K K S G E Y Y K N S L N S L S D K
61 ATAAAAGAC**AAAAAAA**GCGGAGAATACTATAAAAATAGTTTAAATTCTCTGTCAGATAAA

41 Q L L R F K M Y K E K V S P K F F Y L F
121 CAATTATTACGTTTTAAAATGTATAAAGAGAAAGTATCACCAAAGTTTTTTTATCTGTTC

61 L S F T A L M F I L N Y I H L I K L Q N
181 TTATCCTTTACGGCTCTCATGTTCATATTAAATTATATTCATTTAATAAAATTGCAAAAT

81 G L S S V F Y G W K M W I I I V I Y F I
241 GGATTGTCCTCAGTATTTTACGGCTGGAAAATGTGGATAATTATAGTTATTTATTTTATA

101 M N I V L H E L G H I Y S L K F F G K N
301 ATGAATATAGTGCTTCATGAACTGGGACATATTTATTCCTTAAAATTTTTCGGGAAAAAC

121 F D K V G F K L N F Y V F P A F Y V Q L
361 TTTGATAAAGTGGGATTCAAACTGAATTTTTATGTATTTCCTGCTTTTTATGTCCAATTG

141 N E T Y M L S R N E K I I V H L F G L F
421 AATGAAACCTACATGCTATCAAGAAATGAAAAGATAATCGTTCATTTATTTGGCTTGTTT

161 I N Y L L I N T L E L I N Q F T F S S E
481 ATCAATTATCTTTTAATAAATACATTAGAATTAATAAATCAATTTACATTTTCCAGTGAA

181 A L T M A F M L F S S T L L W N L I P I
541 GCATTAACTATGGCATTTATGCTTTTCTCGTCTACTTTATTGTGGAACTTAATCCCAATA

201 L N S D G Y K I L L A F L S L D E Y S R
601 TTGAATTCTGATGGTTACAAAATTCTTTTAGCTTTTCTGAGCTTAGATGAATATAGCCGT

221 F K T N H W L V L T I Q I I G I G L A V
661 TTTAAGACAAATCATTGGTTAGTTCTTACGATTCAAATTATAGGGATTGGCCTTGCAGTG

241 N S V V H W I L Y I V N *
721 AATTCAGTTGTACATTGGATATTATATATTGTAAACTAG

B.

1 M K I L K Y E D E K Y E V L V Q N N V F
1 ATGAAAATTTTAAAGTATGAAGATGAAAAATATGAAGTGCTTGTGCAAAACAACGTATTT

21 I K D K K K A E N T I K I V *
61 ATAAAAGAC**AAAAAAAAA**GCGGAGAATACTATAAAAATAGTTTAAATTCTC...

Figure S4: Nucleotide and corresponding amino acid sequences of *epeP* in isolates of *B. subtilis*. (A) shows the nucleotide (bottom line) and corresponding amino acid (top line) sequences in isolates NCIB 3610, NRS6116, NRS6118, NRS6153, NRS6190 and NRS6202 and (B) shows the same sequences of isolates NRS6107 and NRS6121. The point at which the protein sequence diverges is indicated in red. The asterisk represents a stop codon.

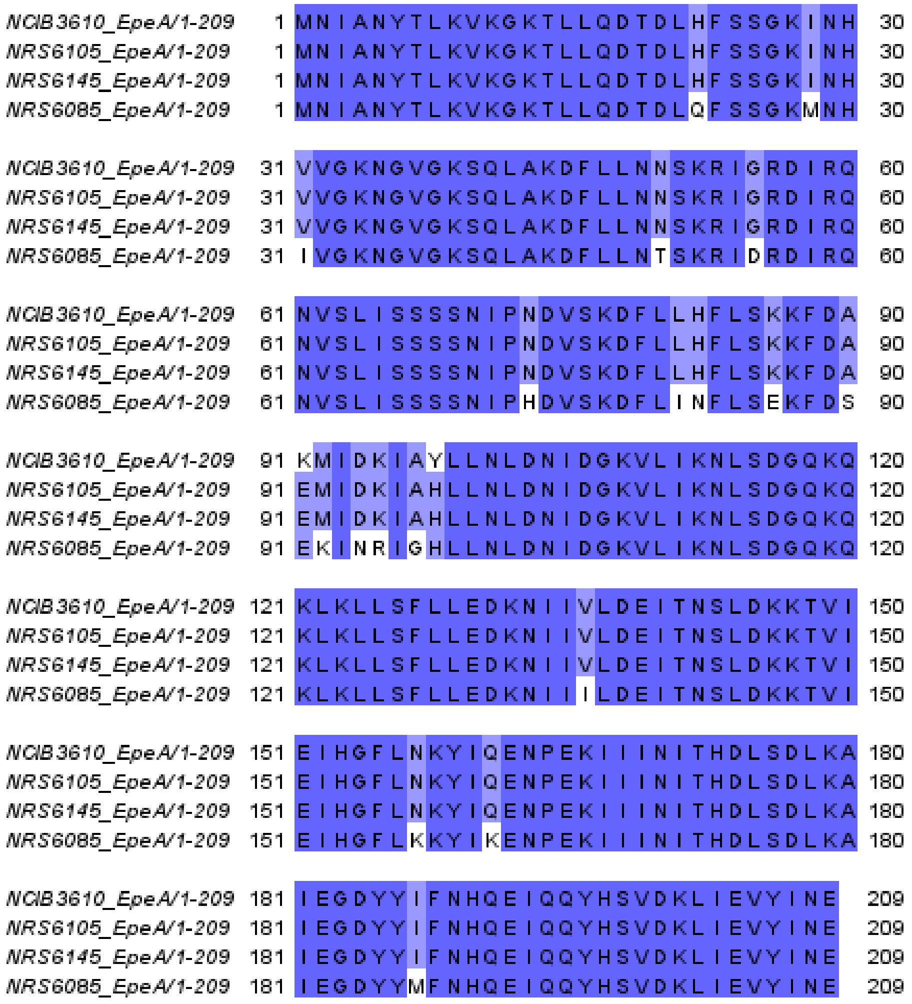

Figure S5: Alignment of EpeA sequences from *B. subtilis* isolates. Sequence alignments were performed in Jalview (3) using Mafft with default settings. The colouring scheme used represents percent identity, where amino acids coloured in dark blue correspond to an identity of >80% to the consensus sequence. The lighter shade of blue represents sequence identities of >60%. Amino acids coloured in white show a sequence identity of less than 40% to that of the consensus sequence.

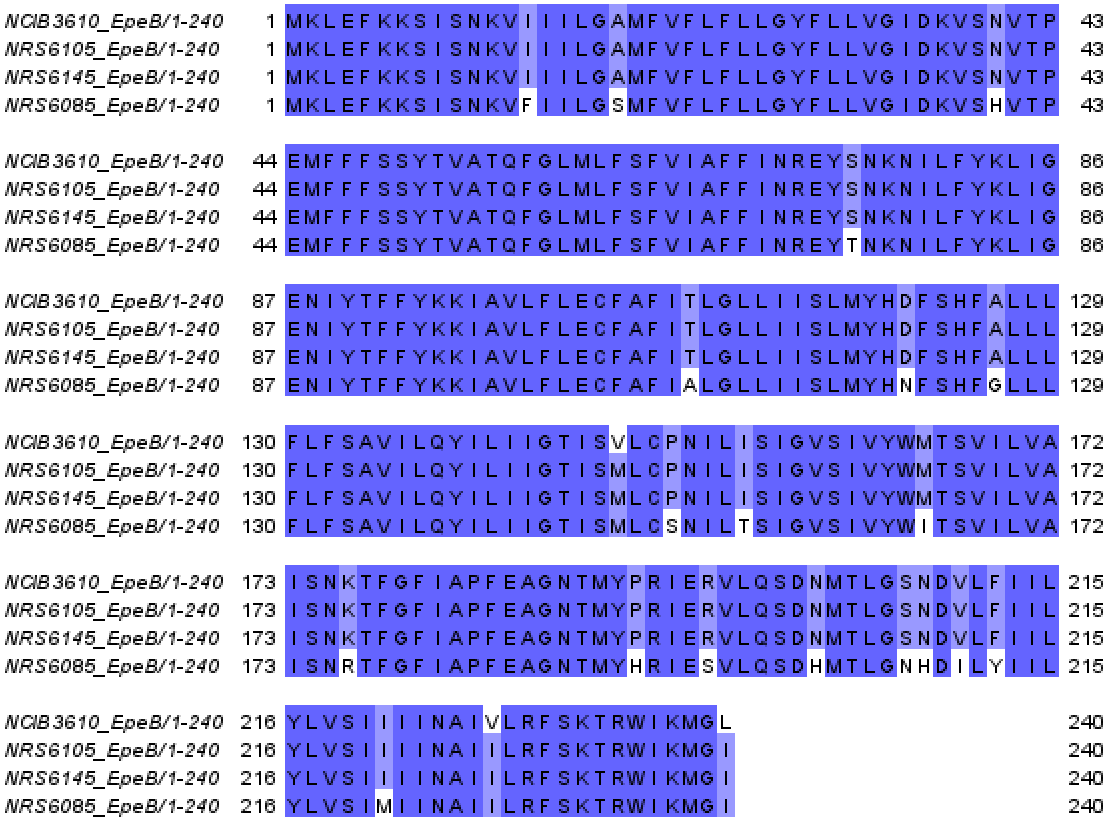

Figure S6: Alignment of EpeB sequences from *B. subtilis* isolates. Sequence alignments were performed in Jalview (3) using Mafft with default settings. The colouring scheme used represents percent identity, where amino acids coloured in dark blue correspond to an identity of >80% to the consensus sequence. The lighter shade of blue represents sequence identities of >60%. Amino acids coloured in white show a sequence identity of less than 40% to that of the consensus sequence.

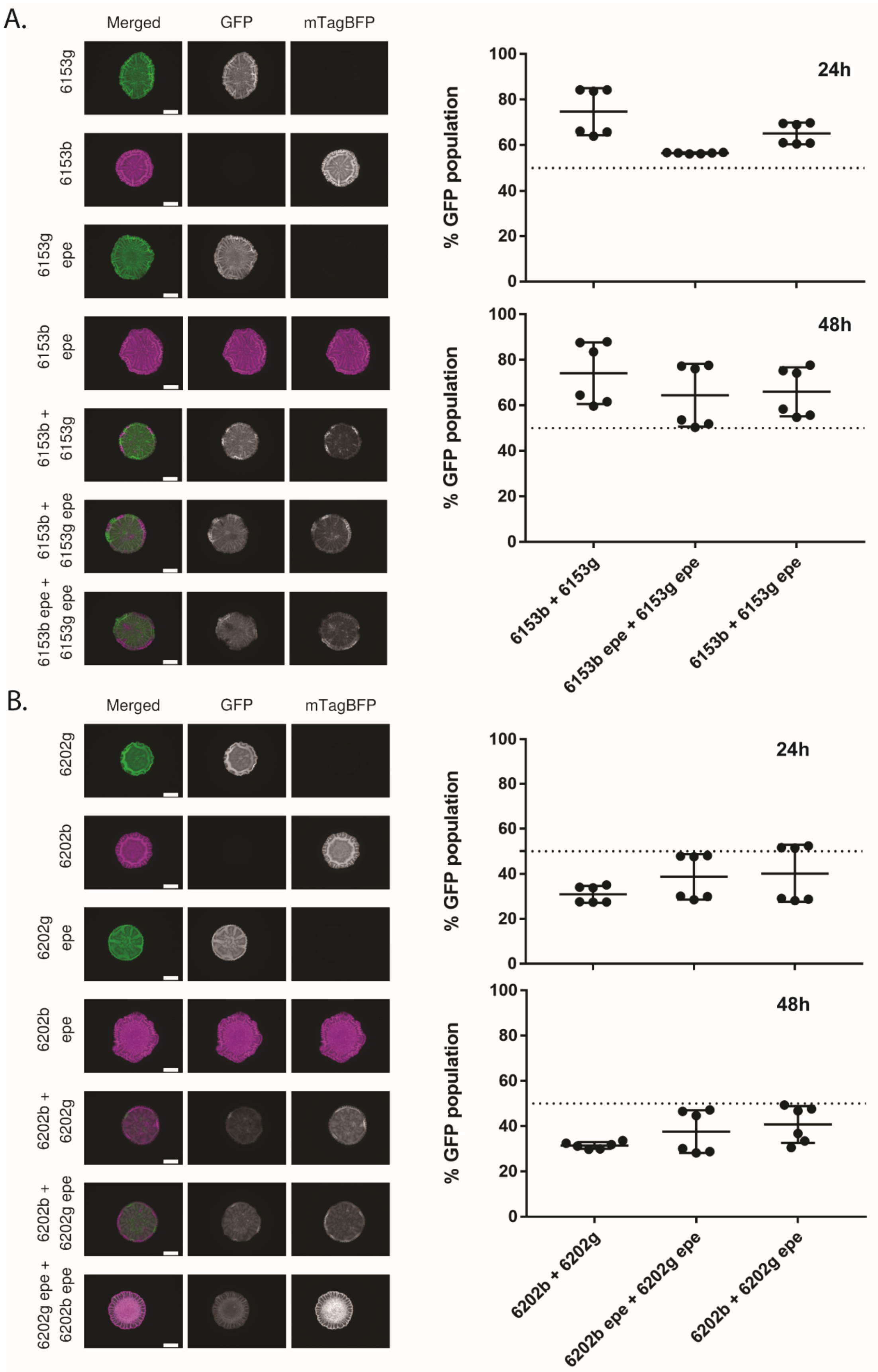
A.
Merged
GFP
mTagBFP
6153g
6153b
6153g epe
6153b epe
6153b + 6153g
6153b + 6153g epe
6153b epe + 6153g epe
% GFP population
24h
48h
0
20
40
60
80
100
6153b + 6153g
6153b epe + 6153g epe
6153b + 6153g epe
B.
Merged
GFP
mTagBFP
6202g
6202b
6202g epe
6202b epe
6202b + 6202g
6202b + 6202g epe
6202g epe + 6202b epe
% GFP population
24h
48h
6202b + 6202g
6202b epe + 6202g epe
6202b + 6202g epe

Figure S7: Competition outcomes of *epeXEPAB* deletion strains of NRS6153 and NRS6202. On the left, representative images of biofilms growth for 48 hours at 30°C on MSgg agar are shown. "6153" is the soil isolate NRS6153 (A) and "6202" is the isolate NRS6202 (B). Strain names followed by "b" represent strains constitutively expressing mTagBFP and names followed by "g" represent strains constitutively expressing GFP. "epe" represents deletion of the *epeXEPAB* operon. The scale bars represent 0.5 cm. On the right, competition results of either NRS6153 (A) or NRS6202 (B) wild type expressing mTagBFP (NRS6938 and NRS7201 respectively) against GFP-expressing wild type (NRS6222 and NRS6897) or *epeXEPAB* mutant (NRS7390 and NRS7392 respectively) of the soil isolates after 24 and 48 of co-incubation on biofilm inducing media plates as indicated. The presented values are the % of the community of GFP expressing strain, quantified using image analysis. Each individual data point presented for each isolate represent one of three technical replicates for the two biological repeats performed. The error bars represent the standard deviation of the mean.

Table S1: Synthetic sequence used to construct the *epeXEPAB* mutants

plasmid	Construct sequence
pNW2315[a]	GGATCCGCGAGTAGATCTAGGTACTTGCTTTCCAAAACCGTCCATCCCCTTTTTAGAATAAACCCCTCTT<br>TATGAGCATGTGCATGATAAGGTGAATTTAAAAGTATGACATTATTTTTAAACATTGGACCTTTATATG<br>ATAGTACCGCATAACTGCTTTTAGAGACAATTAAAACGAGAGAAAGAGGTGCCAAATGGGGTAAGCA<br>AGAGCTTAAAACAGCTTTTCAGTACAGGTTGGTTTAGGGTAAGATTGTTTGCCTCATTTCTTCCACTTA<br>AAATCTGTATATCCCTTAACTAGTGTAGTCAATATTGATGTTTTACTTTTCATTACGAAGCTTTAAATCA<br>AACTATGGCCTTTCATTCGGTATAGCTTAGAGGGGCTAGAGGACTATAGGCTTTATATACCAAGAATA<br>GTGACATTTTTCAGATTTTTTAGGTACAATATATTGACATGTATTGAATGATATAGAATAATTGGTTTAT<br>ATTAGAAAAGGAGGAGGGATAATATGGTTTGATTTTTAATGGATAATGTGATATAATCTTTAAATACT<br>GTAGAAAAGAGGAAGGAAATAATAAATGGCTAAAATGAGAATATCACCGGAATTGAAAAAACTGATC<br>GAAAAATACCGCTGCGTAAAAGATACGGAAGGAATGTCTCCTGCTAAGGTATATAAGCTGGTGGGAG<br>AAAATGAAAACCTATATTTAAAAATGACGGACAGCCGGTATAAAGGGACCACCTATGATGTGGAACG<br>GGAAAAGGACATGATGCTATGGCTGGAAGGAAAGCTGCCTGTTCCAAAGGTCCTGCACTTTGAACGG<br>CATGATGGCTGGAGCAATCTGCTCATGAGTGAGGCCGATGGCGTCCTTTGCTCGGAAGAGTATGAAG<br>ATGAACAAAGCCCTGAAAAGATTATCGAGCTGTATGCGGAGTGCATCAGGCTCTTTCACTCCATCGAC<br>ATATCGGATTGTCCCTATACGAATAGCTTAGACAGCCGCTTAGCCGAATTGGATTACTTACTGAATAAC<br>GATCTGGCCGATGTGGATTGCGAAAACTGGGAAGAAGACACTCCATTTAAAGATCCGCGCGAGCTGT<br>ATGATTTTTTAAAGACGGAAAAGCCCGAAGAGGAACTTGTCTTTTCCCACGGCGACCTGGGAGACAG<br>CAACATCTTTGTGAAAGATGGCAAAGTAAGTGGCTTTATTGATCTTGGGAGAAGCGGCAGGGCGGAC<br>AAGTGGTATGACATTGCCTTCTGCGTCCGGTCGATCAGGGAGGATATCGGGGAAGAACAGTATGTCG<br>AGCTATTTTTTGACTTACTGGGGATCAAGCCTGATTGGGAGAAAATAAAATATTATATTTTACTGGATG<br>AATTGTTTTAGTACCTAGATAATAAAGGGAGCTACTTCACAATGAAGTAGCTTTTTATGTGCACCTATA<br>TATCATTAACATCAAGATTAATGCATGTGACTGGCTGAGTTTGTTAAGGGAAGGATGTTTTTCTGTTTG<br>CATCGCCCAAACTATTATTGTTTTTCTTTCACCATCTTTACAAAGAGGAAAAAGATAATGAATACGATA<br>AAGTAGTTCATTGGATTCTTAACGATGCTCATCTCTAGTGAAATCTGTAAGAGTATCCAGAGCGCAAA<br>GATGGCTAGTATGGCAGGAATGATAATTTTGTACATGTCGACCACCTACCGTAGTGAAGAGCCCTATA<br>ATAAAAAACTATTAGCCTGTACCACAAACTGCGACTGCTCATAATTATACGAAATCTTCGAGTAATCAA<br>ACGGCTGCCCATTTGTCAGGTGAAAAATACTTTCTATATACAGCTTCGGCAGTCCAGCCTCTAATCCCA<br>AATATTCCGCTTCTTCCTCATTAAGCTGGCCCACGTGCCTGCAG

.

[a] nucleotides 7 to 509 correspond to base pairs 4127683 to 4128185 of the NCIB 3610 genome; nucleotides 1,380 to 1,879 are base pairs 4123761 to 4124260 of the NCIB 3610 genome; and nucleotides 510 to 1,379 carry the kanamycin resistance cassette. The construct was inserted into a pUC57 cassette using BamHI/PstI restriction sites.

Table S2: Overview of assembled genomes of *B. subtilis*

Isolate	Accession	Chromosomal Contigs	Plasmids	Length (bp)	N50 (bp)	GC (%)	Annotated CDS	Annotated rRNA	Annotated tRNA
NRS6085	ERZ15916258	1	1	4,342,529	4,335,289	43.4	4,405	30	86
NRS6096	ERZ15916259	2	3	4,312,183	4,128,244	43.4	4,330	30	88
NRS6099	ERZ15916260	1	0	4,096,107	4,096,107	43.8	4,064	30	86
NRS6103	ERZ15916261	1	0	4,264,553	4,264,553	43.5	4,271	30	86
NRS6105	ERZ15916262	1	0	4,090,679	4,090,679	43.8	4,021	30	86
NRS6107	ERZ15916263	1	1	4,166,364	4,101,676	43.7	4,126	30	86
NRS6116	ERZ15916264	1	1	4,371,489	4,307,899	43.3	4,448	27	86
NRS6118	ERZ15916265	1	0	4,261,356	4,261,356	43.5	4,282	30	86
NRS6121	ERZ15916267	1	0	4,123,859	4,123,859	43.8	4,084	30	86
NRS6127	ERZ15916268	1	0	4,226,657	4,226,657	43.5	4,244	30	86
NRS6128	ERZ15916269	1	0	4,001,615	4,001,615	43.9	3,930	30	86
NRS6131	ERZ15916270	1	1	4,257,224	4,249,983	43.2	4,334	30	87
NRS6132	ERZ15916271	1	1	4,181,988	4,097,627	43.6	4,162	30	88
NRS6145	ERZ15916273	1	1	4,169,376	4,085,688	43.6	4,117	27	88
NRS6153	ERZ15916274	1	0	4,215,744	4,215,744	43.7	4,211	30	86
NRS6160	ERZ15916275	1	0	4,224,542	4,224,542	43.6	4,218	30	86
NRS6167	ERZ15916276	1	0	4,192,866	4,192,866	44	4,116	30	86
NRS6181	ERZ15916277	1	1	4,275,379	4,189,561	43.6	4,338	30	88
NRS6183	ERZ15916278	1	0	4,256,610	4,256,610	43.5	4,283	30	86
NRS6186	ERZ15916279	1	1	4,324,000	4,239,628	43.3	4,363	30	88
NRS6187	ERZ15916280	1	0	4,096,109	4,096,109	43.8	4,063	30	86
NRS6190	ERZ15916281	2	1	4,169,151	4,056,987	43.7	4,147	30	86
NRS6202	ERZ15916282	3	0	4,248,983	4,243,504	43.5	4,283	24	86

Type of molecule	Most similar known cluster	% ID	Biosynthetic gene
Ranthipeptide, sactipeptide	Sporulation killing factor	100%	*skfC*
Glycocin	Sublancin 168	100%	*sunA*
RRE-containing			*aslA* (*yxbA*)
CDPS			*yvmC*
Lanthipeptide	subtilomycin	100% / 83%	*subB, subC*
Epipeptide	thailanstatin A	10%	*yydG (epeE), yydF (epeX)*

Table S3: Accessory clusters predicted by antiSMASH v.6.0. RRE stands for RiPP (ribosomally synthesized and posttranslationally modified peptide) recognition element. CDPS stands for tRNA-dependent cyclodipeptide synthases. % ID shows the percent identity of the cluster to the most similar known cluster in the AntiSMASH database and the biosynthetic gene is the core biosynthetic identified gene(s) in the *B. subtilis* genome. Gene names in brackets indicate synonyms of gene names. For the lanthipeptide subtilomycin, the % ID is presented is 100 for all isolates encoding the cluster apart from one (NRS6132), which has a % ID of 83 compared to the rest.

Region	Isolate	% ID	alignment length (bp)	mismatches	gap opens	s. start	s. end	E value
epeX	NCIB 3610	100	150	0	0	4127685	4127536	2.10E-74
epeX	NRS6107	100	150	0	0	4013218	4013069	2.10E-74
epeX	NRS6116	100	150	0	0	4165587	4165438	2.10E-74
epeX	NRS6118	100	150	0	0	1380910	1380761	2.10E-74
epeX	NRS6121	100	150	0	0	270958	270809	2.10E-74
epeX	NRS6153	100	150	0	0	4127374	4127225	2.10E-74
epeX	NRS6190	100	150	0	0	4016655	4016506	2.10E-74
epeX	NRS6202	100	150	0	0	4170601	4170452	2.10E-74
epeE	NCIB 3610	100	960	0	0	4127478	4126519	0
epeE	NRS6107	100	960	0	0	4013011	4012052	0
epeE	NRS6116	100	960	0	0	4165380	4164421	0
epeE	NRS6118	100	960	0	0	1380703	1379744	0
epeE	NRS6121	100	960	0	0	270751	269792	0
epeE	NRS6153	100	960	0	0	4127167	4126208	0
epeE	NRS6190	100	960	0	0	4016448	4015489	0
epeE	NRS6202	100	960	0	0	4170394	4169435	0
epeP	NCIB 3610	100	759	0	0	4126538	4125780	0
epeP	NRS6107	99.474	761	0	2	4012071	4011313	0
epeP	NRS6116	100	759	0	0	4164440	4163682	0
epeP	NRS6118	100	759	0	0	1379763	1379005	0
epeP	NRS6121	99.215	764	0	3	269811	269049	0
epeP	NRS6153	100	759	0	0	4126227	4125469	0
epeP	NRS6190	100	759	0	0	4015508	4014750	0
epeP	NRS6202	100	759	0	0	4169454	4168696	0
epeA	NCIB3610	100	630	0	0	4125630	4125001	0

Region	Isolate	% ID	alignment length (bp)	mismatches	gap opens	s. start	s. end	E value
epeA	NRS6107	100	630	0	0	4011163	4010534	0
epeA	NRS6116	100	630	0	0	4163532	4162903	0
epeA	NRS6118	100	630	0	0	1378855	1378226	0
epeA	NRS6121	100	630	0	0	268899	268270	0
epeA	NRS6153	100	630	0	0	4125319	4124690	0
epeA	NRS6190	100	630	0	0	4014600	4013971	0
epeA	NRS6202	100	630	0	0	4168546	4167917	0
epeA	NRS6145	99.206	630	5	0	3990995	3990366	0
epeA	NRS6105	99.206	630	5	0	3995988	3995359	0
epeA	NRS6085	88.254	630	74	0	2301933	2302562	0
epeB	NCIB 3610	100	723	0	0	4124980	4124258	0
epeB	NRS6107	100	723	0	0	4010513	4009791	0
epeB	NRS6116	100	723	0	0	4162882	4162160	0
epeB	NRS6118	100	723	0	0	1378205	1377483	0
epeB	NRS6121	100	723	0	0	268249	267527	0
epeB	NRS6153	100	723	0	0	4124669	4123947	0
epeB	NRS6190	100	723	0	0	4013950	4013228	0
epeB	NRS6202	100	723	0	0	4167896	4167174	0
epeB	NRS6145	99.032	723	1	0	3990345	3989623	0
epeB	NRS6105	99.032	723	1	0	3995338	3994616	0
epeB	NRS6085	87.866	717	87	0	2302583	2303299	0

Table S4: Results of nucleotide BLAST analysis of the *epeX*, *epeE*, *epeP*, *epeA* and *epeB* coding sequences from *B. subtilis* 168 against the isolates used in this work. "s." in "s. start" and "s. end" strands for "subject" and these values show the coordinate on the chromosome of each subject isolate where the indicated gene sequence starts and ends. % ID is the percent identity of the query sequence to the subject. The alignment length is in base pairs.

Supplemental References

1. Gilchrist CLM, Chooi YH. 2021. Clinker & clustermap.js: Automatic generation of gene cluster comparison figures. Bioinformatics doi:10.1093/bioinformatics/btab007.
2. Blin K, Shaw S, Kloosterman AM, Charlop-Powers Z, van Wezel GP, Medema MH, Weber T. 2021. antiSMASH 6.0: improving cluster detection and comparison capabilities. Nucleic Acids Res doi:10.1093/nar/gkab335.
3. Waterhouse AM, Procter JB, Martin DM, Clamp M, Barton GJ. 2009. Jalview Version 2--a multiple sequence alignment editor and analysis workbench. Bioinformatics 25:1189-91.
